# Disease propagation in amyotrophic lateral sclerosis (ALS): an interplay between genetics and environment

**DOI:** 10.1186/s12974-020-01849-7

**Published:** 2020-06-06

**Authors:** Sarah Schram, Jeffrey A. Loeb, Fei Song

**Affiliations:** Department of Neurology and Rehabilitation, University of Illinois at Chicago, NPI North Bldg., Room 657, M/C 796, 912 S. Wood Street, Chicago, IL 60612 USA

**Keywords:** Nerve injury-mediated neuroinflammation, Neuronal-glial communication through glial growth factor neuregulin, Inhibition of neuregulin-mediated inflammation

## Abstract

Amyotrophic lateral sclerosis (ALS) is a progressive, fatal disease affecting the neuromuscular system. While there have been a number of important genetic discoveries, there are no therapeutics capable of stopping its insidious progression. Lessons from clinical histories reveal that ALS can start focally at a single limb, but then segmentally spread up and down the spinal cord as well as in the motor cortex and cortex of frontal and temporal lobes until respiratory muscles fail. With or without a clear genetic etiology, often there is no explanation as to why it starts in one region of the body versus another. Similarly, once the disease starts the mechanisms by which the neurodegenerative process spreads are not known. Here, we summarize recent work in animal models that support the hypothesis that critical environmental contributions, such as a nerve injury, can initiate the disease process. We also propose that pathological axoglial signaling by the glial growth factor neuregulin-1 leads to the slow propagation of neuroinflammation resulting in neurodegeneration up and down the spinal cord and that locally applied drugs that block neuregulin-1 signaling could slow or halt the spread of disease.

## Introduction

Amyotrophic lateral sclerosis (ALS) is a neurodegenerative disease characterized by spreading paralysis which can originate in any part of the body. The disease is poorly understood with no effective therapeutics and an average survival time of less than 5 years according to the ALS association. Given the wide variations in genetic links and variable clinical presentations even with the same genetic mutations, environmental contributions are likely to be very important in ALS [1]. Veterans and athletes have been shown to be at an increased risk of developing the disease, suggesting injury may act as an environmental trigger [2–4]. Here, we discuss potential mechanisms that contribute to disease progression, the possibility of injury as an instigating event, and the potential role of the growth factor neuregulin in disease spread.

## Environmental contributions

It has long been suggested that environmental factors such as lead, pesticides, trauma, and physical activity can act as triggers for ALS [5]. Population studies show that the disease is 2–3 times more common in varsity and professional athletes and veterans, even without combat experience [2–4, 6–8]. In fact, heightened physical activity in general appears to be correlated with a greater risk of disease [4, 9]. One potential explanation is that focal nerve injury could trigger disease onset in a specific limb. Head trauma may also play a role based on both animal and patient studies [10, 11]. Despite the multiple case studies and larger epidemiological studies suggesting a link, other studies have shown conflicting results [12, 13]. This indicates that any role injury may play is complicated and probably includes a mosaic of other predispositions including genetic susceptibility, gender, age, type, and location of injury. Furthermore, a study in Denmark, showed that timing of the injury (more than 5 years prior to diagnosis) and age of injury (before age 55) is crucial for the degree of increased risk and may explain some confounding results in prior studies [14]. Unfortunately, without the availability of definite biomarkers, the contribution of injury remains challenging to prove.

## Animal models combining nerve injury with genetics

Animal models have been useful in investigating the effects of injury on disease development and progression in ALS models. A study by Sharp and colleagues demonstrated that in the mutant superoxide dismutase 1 (SOD1)-expressing mouse, a sciatic nerve crush induced changes in fatigue-resistance characteristics and muscle fiber type in the extensor digitorum longus muscle prematurely at times when deficits are not normally seen. This injury also increased motor neuron loss in the ventral horn of the spinal cord [15]. Another study showed similar findings following facial motor neuron damage [16].

**Fig. 1:**
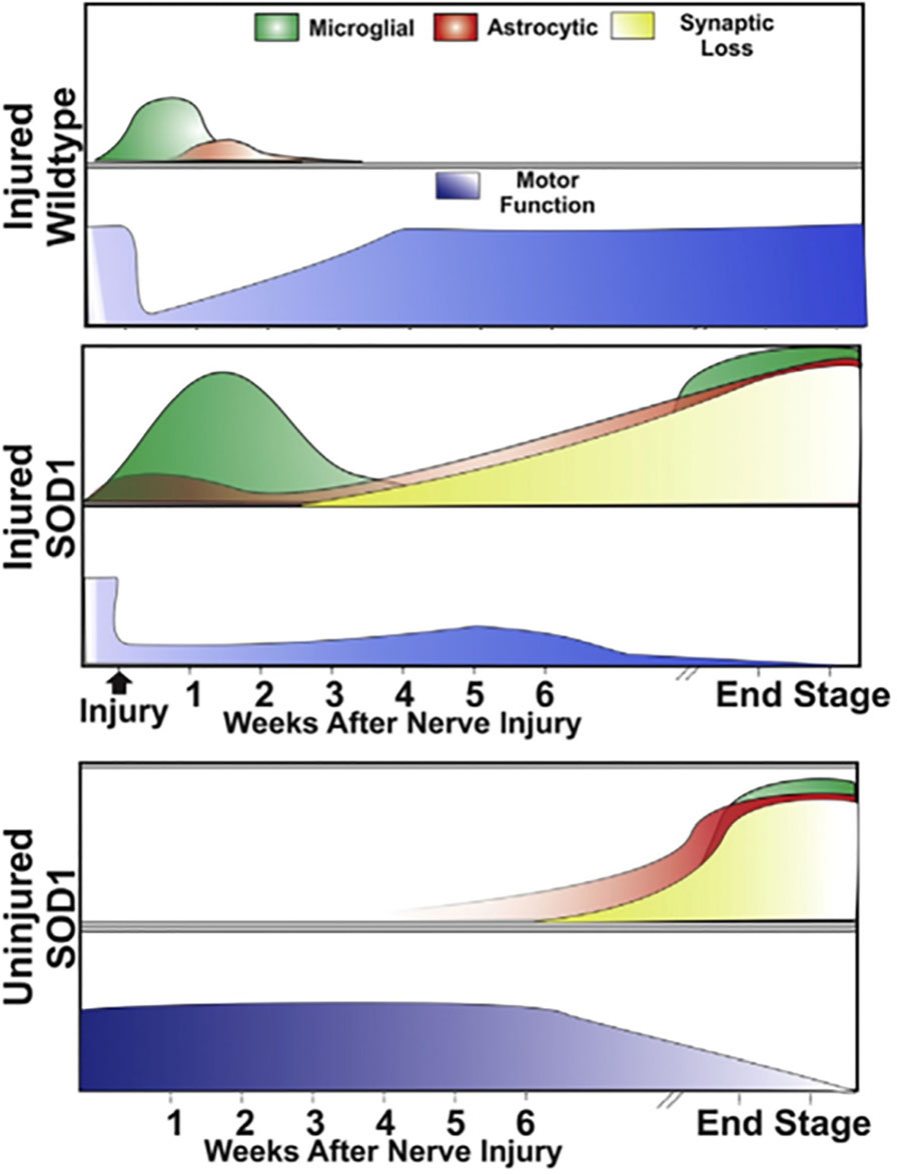
Time course of injury-induced functional, inflammatory, and neuronal changes in the SOD1 rat. This figure summarizes the longitudinal effects of mutant SOD1 protein both on motor function and cellular pathophysiology after a single sciatic nerve injury. While WT animals recovered fully from the injury within 5 weeks, they had only mild microgliosis/astrocytosis in the acute recovery stage. In contrast, injured SOD1 animals failed to recover and showed increased and sustained microgliosis followed by a premature astrocytic recruitment and neuronal synaptic loss. This model combines an environmental insult with a genetic defect that can help elucidate the functional and physiological effects of ALS disease onset and progression that could be used to develop targeted therapeutics (taken from [18] with the permission)

The mutant SOD1-expressing rat has also been used in similar experiments. Unlike mice, rats with SOD1 mutations have shown variability in the site of symptom onset. Whereas mice consistently present with initial lower limb weakness, rats can present with lower limb or upper limb involvement [17]. A recent study by Schram et al. [18] investigated the effects of a sciatic nerve crush on disease progression and inflammation in the SOD1 rat. A single, unilateral crush lesion of the sciatic nerve at 10 weeks of age, prior to disease onset, hastened functional decline and shortened survival compared to uninjured SOD1 littermates [18]. Whereas control animals regained full motor function within a few weeks following injury, the SOD1 rats never recovered and developed weakness on the contralateral leg well before other, non-injured SOD1 rats. This suggests that the single nerve injury initiated an earlier disease onset and promoted the local spread of disease. Quantitative histological studies on the spinal cord at different time points coinciding with decreases in motor function demonstrated a markedly increased and prolonged microglial inflammatory response followed by earlier and more pronounced astrocytic recruitment in the SOD1 animal versus controls (Fig. 1). This localized, increased inflammatory response was associated with a significant reduction in motor neuron synaptic connectivity that could explain how the heightened, injury-induced inflammatory response leads to earlier motor neuron degeneration [18].

Some of these effects may be mediated by activity, as another study showed hyperstimulation of the phrenic nerve via implanted electrode in the SOD1 rat showed more rapid motor neuron loss, denervation of the diaphragm muscle, and decreased life span [19]. Interestingly, this stimulation also led to spread of disease-associated weakness to the forelimbs, altering disease progression from that typically seen in SOD1 rodents. In contrast, other studies show a paradoxical effect, where a tibial nerve crush injury in an SOD1 rat at the L5 spinal nerve or distal sciatic nerve led to an increase in motor neuron survival in a subset of motor neurons following disease onset [20, 21]. However, these studies showed no effect on time to disease onset, duration, or survival.

## Inflammatory response to injury as a driver of neurodegeneration

The normal injury response is composed of a series of tightly regulated cellular and molecular processes involving numerous signaling cascades designed to aid recovery [22]. Additionally, the degree and duration of the injury response can vary based on severity and location. In general, there are two phases of injury recovery, acute and chronic, and each is associated with changes in gene transcription and translation in a variety of cell types. As part of the injury repair process, microglia are thought to strip synaptic connections, as observed in the above nerve injury model in SOD1 rats [18]. Microglia are also known to modify synaptic circuits during development, and more recent studies show a similar process in adult neurogenesis [23, 24]. As part of this experience-dependent modification system, there is extensive contact between microglial processes and synapses. It has been proposed that this contact leads to synaptic rewiring and removal following traumatic events, such as stroke or axonal damage, in which microglial contact increases and synaptic activity decreases [25–27]. Furthermore, microglia express C1q, a major component of the complement system and inducer of phagocytosis. C1q-tagged synapses have been described in the brain and suggested to be a mechanism for synaptic removal [28, 29].

In ALS, microglia appear to play a highly dynamic role as their phenotypic expression changes as the disease progresses. Microglia secrete neuroprotective factors such as IL-10 in pre-symptomatic stages, then adopt a more detrimental state as disease onset occurs and secrete activators associated with inflammation and cell death [30–32]. In vitro studies of primary microglia isolated from neonatal SOD1 mice show an increased response to LPS in terms of cell surface protein expression and changes in size/morphology [33]. Relatedly, microglia collected from the spinal cord of the SOD1 (G93A) rats at three different points in disease progression (presymptomatic, symptom onset, and end-stage) show different activation patterns, suggesting an active role in disease progression [34]. Microglia carrying mutations are also shown to exacerbate and speed up disease progression, while WT microglia promote survival [35–37]. This may be due to heightened inflammatory activity of mutant microglia as well as a pathological response to the presence of misfolded protein [38–40].

Consistently, co-cultures of microglia with motor neurons have shown that microglia can directly lead to motor neuron death and require nuclear factor kappa-light-chain-enhancer of activated B cells (NF-κB) signaling [40]. Together, these studies highlight a crucial role of microglial-dependent inflammation in disease onset and progression and highlight the potential importance of the augmented inflammatory responses observed to occur after injury.

One issue that continues to plague ALS research is the lack of effective therapeutic translation from animal models to patients. Key to improving this track record is identifying shared targets between animal models and the human disorder. Patient studies confirm the presence of abnormal inflammation and microglial activation in post-mortem human tissues [41, 42]. This is in addition to experiments that detected inflammation in the cortex of living ALS patients with positron emission tomography (PET) [43]. Therapeutic targeting of nervous system inflammation continues to be an attractive strategy for ALS treatment, despite having not yet been proven effective in human clinical trials [31]. It is increasingly clear that understanding how and when to reduce inflammatory activation will be important for the treatment of this and many other neurological diseases [34, 44].

## Neuron-glia signaling as a mechanism of disease spread

Neurons have numerous signaling mechanisms to communicate with surrounding glia and other cells. One such signaling factor well studied in development is neuregulin-1 (NRG1). In the nervous system, NRG1 is a predominantly neuron-derived growth and differentiation factor that signals from neurons to surrounding cells through activation of the epidermal growth factor (EGF)-family of tyrosine kinase receptors human epidermal growth factor receptor (HER)2-4 [45–51]. NRG1 supports neuromuscular junction formation during development and has been implicated in a number of disease conditions [52–54]. NRG1, previously called glial growth factor, is a potent mitogen for glial cells and plays a role in nerve injury repair [55] with increased HERs and NRG1 in the initial stages following injury [56, 57]. Specifically, NRG1 expression and release increases during re-innervation of target muscles after injury and promotes remyelination of spinal motor neurons [58].

However, prolonged or excessive NRG1 signaling can be problematic. For example, after peripheral nerve injury in animal models of chronic pain, NRG1 from dorsal root ganglia neurons is released into the spinal cord leading to microglial activation. Blocking NRG1 after nerve injury using a number of agents dramatically reduces microglial activation and the development of chronic pain [59]. This pathological inflammation is thought to contribute to the development of chronic pain through microglia-mediated synaptic rewiring in the dorsal horn of the spinal cord [59, 60]. These observations have led to the exploration of NRG1’s possible role in promoting excessive inflammation in the ventral horn of the spinal cord in ALS. Because of its ability to remain localized within the extracellular matrix through a unique heparin-binding domain, NRG1 accumulation could mediate the slow spread of neuroinflammation and possibly disease spread up or down the spinal cord in ALS.

## Neuregulin signaling is increased in ALS and is a potential therapeutic target

**Fig. 2:**
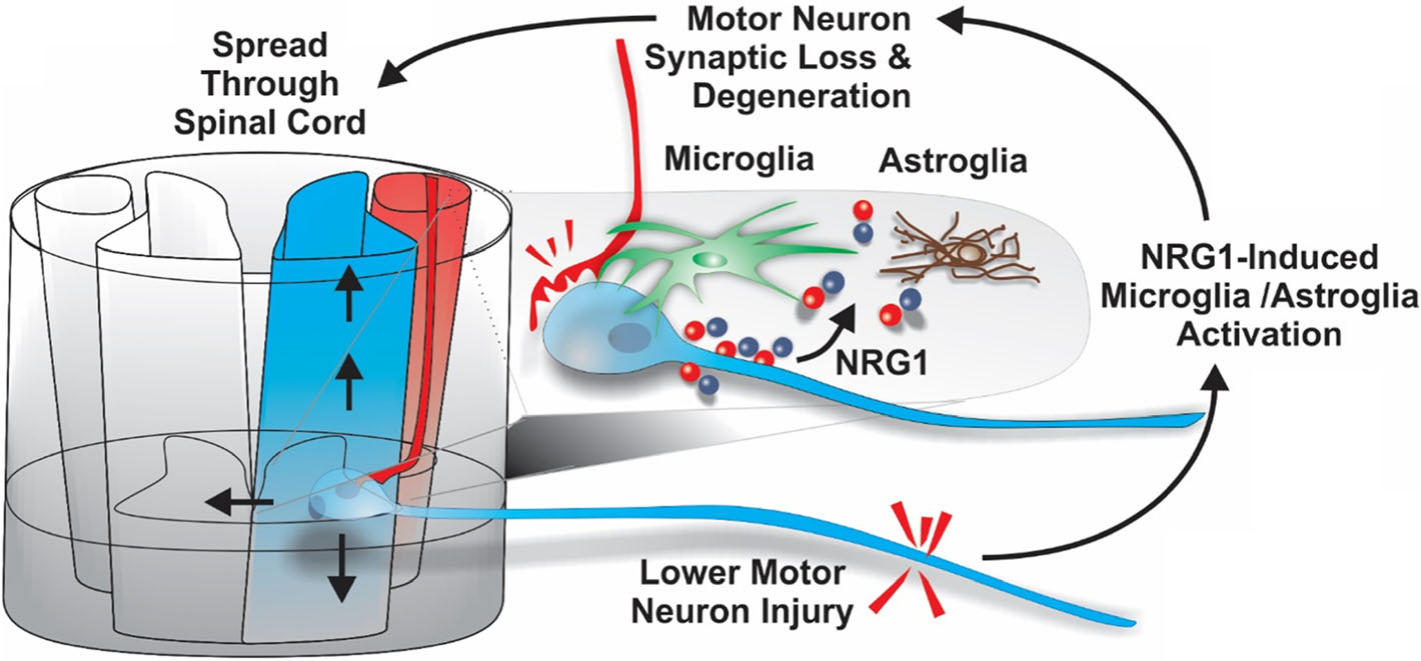
Proposed mechanism of nerve-injury-induced activation of neuregulin-1 leads to spread of disease. In the presence of mutant SOD1 protein, nerve injury produces a heightened, NRG-induced inflammatory response leading to a permanent loss of synapses that spreads up and down the spinal cord and resulting in progressive neurodegeneration. Results summarized in Fig. 1 suggest that this inflammatory response starts with microglia followed by a second wave of astroglial reactivity that correlates with a significant loss of motor neuron synaptic inputs

In ALS, aberrant NRG1 signaling has been shown in human tissues and animal models [42, 61–63]. Specifically, phosphorylated (activated) NRG1 receptors have been observed on activated microglia in the spinal cords of both ALS patients and animal models [41, 42]. This activation, just as in the chronic pain models discussed above, could lead to enhanced inflammation leading to motor neuron damage and death. Neuronal degeneration leads to more NRG1 release producing further microglial activation hence propagating the neurodegenerative process up and down the spinal cord (Fig. 2). Consistently, a highly selective NRG1 antagonist was shown to decrease microglial activation, reduce motor neuron loss, delay disease onset, and prolong survival in the SOD1 G93A mouse model [61]. This antagonist is a humanized fusion protein consisting of the extracellular domain of the HER4 NRG1 receptor and NRG1’s own heparin-binding domain, which, unlike other biologics such as antibodies, allows the drug to penetrate the CNS and directs to the same sites that NRG1 goes [64]. In addition to being humanized and producing no toxic side effects, a key advantage of using this fusion protein to block neuroinflammation is that it can be delivered directly to the brain through the cerebral spinal fluid [60]. Direct CNS delivery has a key advantage of reducing peripheral, off-target effects that have led to significant side effect from other anti-inflammatory drugs given systemically [63]. These features make it an attractive and potentially translatable treatment to stop or slow disease progression in ALS patients.

Delivery of the NRG1 antagonist directly into the central nervous system (CNS) is important in light of other findings suggesting that NRG1 may still be needed to maintain neuromuscular junctions and can be protective in ALS animal models [65–67]. Lasiene et al. [65] reported that viral-mediated delivery of type I-NRG1 (soluble form of NRG1) to the spinal cord had no beneficial effect on disease onset and survival in SOD1 G93A mice. Interestingly, a similar study examining the effects of overexpressing type III-NRG1 in the spinal cord through viral delivery resulted in preserved neuromuscular function of the hindlimbs, improved locomotor performance, and increased numbers of surviving motor neurons along with reduced glial reactivity, but only in female mice [68]. However, viral-mediated delivery of type III-NRG1 (membrane-bound form of NRG1) to the spinal cord restored C-bouton puncta and extended survival in SOD1 mice with no effect on disease onset, suggesting supplementation of membrane-bound NRG1 confers neuroprotection in motor neuron disease [65]. While we similarly showed that intraventricular delivery of NRG1 had no effects on disease onset and survival in SOD1 G93A mice, blocking endogenous NRG1 with an intraventricular antagonist was protective [61]. Taken together, the mode and location of delivery appears to be critical in order to block NRG1 within the CNS, while allowing its normal function in the peripheral nervous system (as highlighted in Fig. 2), to reduce neuroinflammation and neurodegeneration in ALS.

## Conclusions

In summary, inhibition of NRG1-mediated neuroinflammation could prevent synaptic stripping and other toxic effects of chronic inflammation leading to disease progression in ALS, regardless of the underlying genetics. Our studies in the SOD1 G93A mouse model treated with a NRG antagonist support this theory and show an increase in survival [61]. New animal models are needed that better mimic disease progression in humans. Models that use nerve injury to initiate focal and early disease onset could be used to test therapeutics targeting disease progression in a number of genetic models. Another critical area of research needed to advance therapeutics that focus on disease progression will be the identification of reliable and non-invasive biomarkers of disease progression. These key facets will enable a better understanding of disease pathology and paradigm shifts in treatment, ultimately resulting in therapies truly capable of providing hope for patients.

## Data Availability

Not applicable.

## Abbreviations

ALS: Amyotrophic lateral sclerosis
SOD1: Superoxide dismutase 1
NF*-*κB: Nuclear factor kappa-light-chain-enhancer of activated B cells
PET: Positron emission tomography
NRG1: Neuregulin-1
EGF: Epidermal growth factor
HER: Human epidermal growth factor receptor
CNS: Central nervous system
